# Segmentation and classification of medical images using texture-primitive features: Application of BAM-type artificial neural network

**DOI:** 10.4103/0971-6203.42763

**Published:** 2008

**Authors:** Neeraj Sharma, Amit K. Ray, Shiru Sharma, K. K. Shukla, Satyajit Pradhan, Lalit M. Aggarwal

**Affiliations:** School of Biomedical Engineering, Institute of Technology, Banaras Hindu University, Varanasi (UP), India; 1 Department of Computer Engineering, Institute of Technology, Banaras Hindu University, Varanasi (UP), India; 2 Department of Radiotherapy and Radiation Medicine, Institute of Medical Sciences, Banaras Hindu University, Varanasi (UP), India

**Keywords:** Artificial neural network, medical images, segmentation, texture features, texture primitive

## Abstract

The objective of developing this software is to achieve auto-segmentation and tissue characterization. Therefore, the present algorithm has been designed and developed for analysis of medical images based on hybridization of syntactic and statistical approaches, using artificial neural network (ANN). This algorithm performs segmentation and classification as is done in human vision system, which recognizes objects; perceives depth; identifies different textures, curved surfaces, or a surface inclination by texture information and brightness. The analysis of medical image is directly based on four steps: 1) image filtering, 2) segmentation, 3) feature extraction, and 4) analysis of extracted features by pattern recognition system or classifier. In this paper, an attempt has been made to present an approach for soft tissue characterization utilizing texture-primitive features with ANN as segmentation and classifier tool. The present approach directly combines second, third, and fourth steps into one algorithm. This is a semisupervised approach in which supervision is involved only at the level of defining texture-primitive cell; afterwards, algorithm itself scans the whole image and performs the segmentation and classification in unsupervised mode. The algorithm was first tested on Markov textures, and the success rate achieved in classification was 100%; further, the algorithm was able to give results on the test images impregnated with distorted Markov texture cell. In addition to this, the output also indicated the level of distortion in distorted Markov texture cell as compared to standard Markov texture cell. Finally, algorithm was applied to selected medical images for segmentation and classification. Results were in agreement with those with manual segmentation and were clinically correlated.

## Introduction

In recent years, considerable efforts have been made in computer-aided diagnosis (CAD) using medical images to improve a clinician's confidence in the analysis of medical images. Evaluation of medical images by a clinician is qualitative in nature and may vary from person to person. A lot of research efforts have been directed in the field of ‘Medical Image Analysis’ with the aim to assist in diagnosis and clinical studies.[1] Computer-aided analysis of medical images obtained from different imaging systems such as MRI, CT scan, ultrasound B-scan involves four basic steps: a) image filtering or preprocessing, b) image segmentation, c) feature extraction, and d) classification or analysis of extracted features by classifier or pattern recognition system. The image filtering or preprocessing depends mainly on the quality of the image acquired from image acquisition system. The main aim of image preprocessing is to suppress unwanted noise and to enhance image features important from further analysis point of view, and is most of the time specific in nature depending upon the type of noise present in the image. (For example, in case of image with poor ‘brightness and contrast,’ histogram equalization can be used to improve the brightness and contrast of an image.) In analysis of medical images, we try to avoid image preprocessing unless and until it is very much necessary as image preprocessing typically decreases image information content.

Segmentation is an important step in image analysis. Segmentation is a process of dividing an image into regions having similar properties, such as gray level, color, texture, brightness, contrast, etc. The techniques available for segmentation of images can be broadly classified into two classes: I) based on gray level — a) amplitude segmentation methods based on histogram features,[2] b) edge-based segmentation, c) region-based segmentation;[3,4] and II) based on textural feature.[5]

For some typical applications, particularly in the medical image processing, segmentation based on gray level does not give the desired results; in such applications, segmentation based on textural feature methods gives more reliable results; therefore, texture-based analysis is extensively used in analysis of medical images.[6–9] Image analysis based on texture feature of an image is still a complex and challenging problem, and hence texture feature based technique is the approach we have selected for analysis of medical images. Texture can be defined as something consisting of mutually related elements. Further, texture can be defined as spatial arrangement of texture primitives or texture element, sometimes also called textone, arranged in more or less periodic manner, where texture primitive is a group of pixels representing the simplest or basic subpattern. A texture may be fine, coarse, smooth, or grained, depending upon its tone and structure, where tone is based on pixel intensity properties in primitive while structure is the spatial relationship between primitives.[10]

### Feature extraction and classification

This problem has been broadly dealt under the subject area of ‘Pattern Recognition.’ The main aim of pattern recognition is the classification of patterns and subpatterns in an image or scene. A pattern recognition system includes the following subsystems: subsystem to define pattern/texture class, subsystem to extract selected features, and subsystem for classification known as classifier.[11] The pattern classes are normally defined in supervised mode; after this, the desired features are extracted by feature-extracting subsystem, and finally classification is done on the basis of extracted features by classifier. The three main approaches of pattern recognition for feature extraction and classification based on the type of features are as follows: 1) statistical approach, 2) syntactic or structural approach, and 3) spectral approach. In case of statistical approach, pattern/texture is defined by a set of statistically extracted features represented as vector in multidimensional feature space. The statistical features could be based on first-order, second-order, or higher-order statistics of gray level of an image. The feature vector so generated from patterns is assigned to their specific class by probabilistic or deterministic decision algorithm.[12] In case of syntactic approach, texture is defined by texture primitives, which are spatially organized according to placement rules to generate complete pattern. In syntactic pattern recognition, a formal analogy is drawn between the structural pattern and the syntax of language.[13] In case of spectral method, textures are defined by spatial frequencies and are evaluated by autocorrelation function of a texture. A brief survey of methods available for textural feature extraction and classification based on the above approaches is as follows: co-occurrence matrix method based on statistical description of gray level of an image,[14,15] gray level run length method,[16] fractal texture description method,[17] syntactic method,[18] Fourier filter method.[19] As a comparison between the above-mentioned three approaches, spectral frequency–based methods are less efficient, while statistical methods are particularly useful for random patterns/textures; while for complex patterns, syntactic or structural methods give better results.

Segmentation and classification can be achieved as is done in human vision system, which recognizes objects; perceives depth; identifies different textures, curved surface, or a surface inclination by texture information and brightness information collectively called as textone.[20] Therefore, the present approach is hybridization of syntactic and statistical approaches for texture-based segmentation and classification with artificial neural network (ANN) as segmentation and classifier tool. In this scheme, we have used first- and second-order statistical features of the texture-primitive cell for segmentation and classification. In contrast with the syntactic approach, instead of using rules and grammar to represent pattern in terms of sentences, we used analysis by synthesis method.[21] The image is re-synthesized on the basis of classification data produced by ANN. The location and size of texture to be segmented and classified can be directly seen.

## Materials and Methods

We have designed and developed an algorithm for analysis of medical images based on hybridization of syntactic and statistical approaches, using artificial neural network (ANN).

The textural properties are derived by using the 1) first-order statistics and 2) second-order statistics that are computed from spatial gray-level co-occurrence matrices (GLCMs). The data is of normalized type; the data so generated contains normalized feature vectors computed around each pixel. The normalized feature vector contains altogether 17 features computed over the window size of ‘n×n’ pixel matrix. The feature vector includes three features derived from first-order statistics: (i) mean, (ii) standard deviation, and (iii) entropy; and 14 features derived from second-order statistics: (i) contrast, (ii) angular second moment, (iii) inverse difference moment, (iv) correlation, (v) homogeneity, (vi) variance, (vii) cluster tendency computed over distance 1 and 2 respectively. (The details of these features and their computation scheme are presented in the ‘Algorithm’ section.) The data set is presented to a bidirectional associative memory (BAM)-type artificial neural network[22] for segmentation and classification of the images. BAM network classifier has guaranteed convergence and stronger error-correction probability with less connection complexity compared with other neural networks. Further, BAM-type ANN performs function similar to the human brain property of association and can recall regions of interest (ROI) even in the presence of artifacts. BAM-type ANNs were introduced by Kosko,[23] which are two-level nonlinear neural networks. In this algorithm, BAM-type ANN has been specifically modified for segmentation and classification of medical images. Regarding the filtering of images prior to segmentation, we would like to state that BAM-type artificial neural networks are capable of recognizing patterns even in the presence of noise and have excellent error-correction capabilities;[22] although it is always desirable to filter the image, provided the type of noise and degradations in image are known in advance. Hence in the present work, we have not done any prior filtering.

### Algorithm

**Figure 1:**
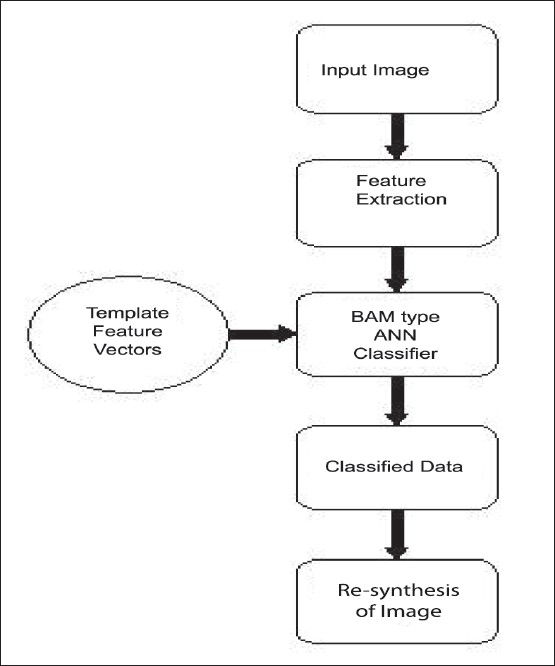
System configuration

The configuration for the proposed system is shown in Figure 1.

The BAM-based algorithm modified for processing gray scale images, i.e., pixels having real coded values, is as follows:

The strategy for segmentation can be explained through the following steps:

- First step: The region of interests to be segmented is required to be identified and labeled. The region of interests (ROIs) could be lesion, tumor, normal tissue, bone, or any other relevant matter; for example, it could be white matter, gray matter, cerebrospinal fluid, hemorrhages site, etc. Further, the type of ROIs to be segmented depends upon the type of application in which user is interested.
- Second step: After specifying the ROI, sample of template is selected by placing the template selection window over the same ROI.
- Third step: The texture features (comprising of both first- and second-order statistical features) of the selected template primitiveare calculated.
- Fourth step: Texture features are calculated around each pixel in the specified neighborhood of the image to be segmented for selected ROIs.
- Fifth step: BAM-type ANN then classifies the pixels corresponding to the features of ROIs as specified, in a supervised manner, in step 2.
- Sixth step: Based on the pixels classification, the segmented image is reconstructed.

### Step 1

The primitive texture cells of the objects to be detected in an image are defined and labeled in a supervised manner. The method for selection of template is simple and straight; the template is selected by placing the template selection window of size ‘n×n’ over the part to be segmented. Pi is the matrix of primitive texture cell of size ‘n×n’ corresponding to the ith ROI to be segmented from the image and is defined in a supervised manner. $\mathrm{Pi}=\begin{array}{cccccccc} {}[ & P_{0,0} & P_{0,1} & & ... & & P_{0,n}; & \\ & P_{1,0} & P_{1,1} & & ... & & P_{1,n}; & \\ & P_{n-1,0} & P_{n-1,1} & & ... & & P_{n-1,n-1}; & {]}_{n\times n} \end{array}$ where pixels are real valued on the scale of 0 to 1.

### Step 2

Extraction of feature vector of texture primitive

First-order features computed for primitive texture cell

- Mean: It is a measure of brightness, μp=1n2∑r=0n-1∑s=0n-1pr,s, where P_r,s_ is pixel at location(r,s).
- Standard deviation: It is a measure of contrast, σp=1n2∑r=0n-1∑s=0n-1pr,s-μp21/2
- Entropy: It is a measure of randomness, e=-∑b=0L-1p(b)log2p(b) , where p(b) N(b)/n^2^ for {0 ≤ b ≤ L-1},

where L is the number of different values which pixels can adopt,

N(b) = number of pixels of amplitude (b) in the pixel window of size ‘n×n’.

Second-order statistical features based on gray-level co-occurrence matrices (GLCMs) computed for primitive texture cell.

**Figure 2:**
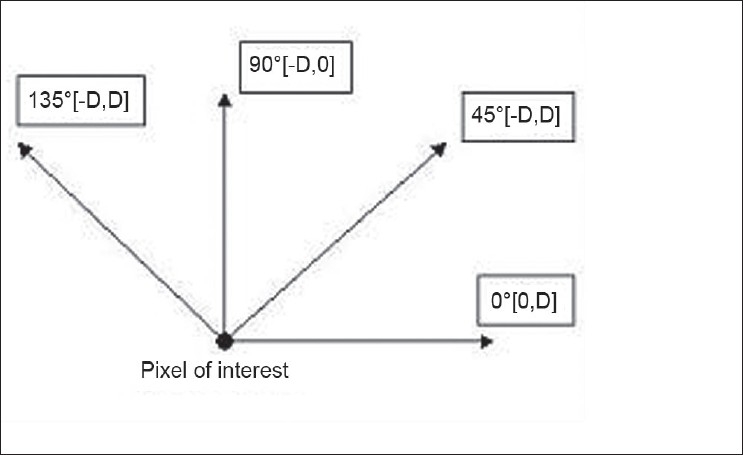
Directionality used in calculating GLCM (D is the distance between two pixels)

The co-occurrence matrix characterizes the spatial interrelationships of the gray tones in an image. The values of the co-occurrence matrix elements present relative frequencies with which two neighboring pixels separated by distance d and at angle θ appear on the image [Figure 2], where one of them has gray level i and other j, and their joint probability of occurrence is given by Pi j.

GLCM features are extracted for ‘n×n’ primitive template matrix in the directions 0°, 45°, 90°, and 135°, and then averaging is done to make them direction invariant. In the present application, GLCM features have been calculated at distances 1 and 2 respectively. Following features are used in the present algorithm.

- Contrast: Contrast is defined as Contrast=∑n=0Ngn2 where n=∑i=1Ng∑j=1NgPi,j·|i-j
  ^Ng^ = number of gray levels.

This measure provides evidence of how sharp the structural variations in the image are.

- Angular second moment: The angular second moment gives a strong measure of uniformity and can be defined as Angular second moment=∑i∑jP2i,j

Higher nonuniformity values provide evidence of higher structural variations.

- Inverse difference moment: Inverse difference moment is the measure of local homogeneity and is defined as Inverse difference moment=∑i∑j11+i-j2Pij
- Correlation: The correlation feature is a measure of gray-level linear dependency of the image.
  Correlation feature is defines as $\mathrm{Correlation}=\frac{\sum_{i}\sum_{j}(i,j)P_{i,j}-\mu^{2}}{\sigma^{2}}$ ,

where *µ* and σ are the mean deviation and standard deviation of the co-occurrence matrix respectively.

- Homogeneity: Homogeneity is the measure that increases with less contrast in the window. It is defined as Homogeneity=∑i∑jPi,j1+|i-j|
- Variance (sum of squares): Variance is the measure that tells us by how much the gray levels are varying from the mean. Variance=∑i∑j(i,j)Pi,j-μ2
- Cluster tendency: This measure indicates, into how many clusters the gray levels present in the image can be classified. Cluster tendency=∑i∑j(i+j-2μ)Pi,j

### Step 3

Normalization of primitive texture cell feature vector is done. $P_{i}=P_{i}/\mathrm{norm}(P_{i})$ where *norm*(*P_i_*) = ‖P_i_‖ = maximum singular value of(P_i_)

### Step 4

Feature vector around each pixel in the local neighborhood of 3×3 of the medical image to be segmented and classified is computed in the same way as defined in step 2. The *P* dimensional feature vector (in present case, the value of *P* = 17) consisting of first- and second-order feature around each pixel is represented as follows: x1=[X1,1  X1,2......................X1,p] x2=[X2,1  X2,2......................X2,p] xn=[Xn,1  Xn,2......................Xn,p]

### Step 5

BAM-based ANN classifies and segments the feature vector of each pixel of image. Generalized architecture and algorithm of BAM-type ANN is given in Appendix-I.

### Step 6

The image is re-synthesized based on the classification data corresponding to the value of (output data); to this, a threshold function is applied. The threshold value of 0.5 corresponding to the 50% desired level of confidence is selected (any other value of threshold can also be selected corresponding to the desired level of confidence) and hence segmentation and classification of soft tissues is achieved.

The software for this algorithm has been developed in MATLAB for segmentation and classification.

## Results and Discussion

The experimental testing of algorithm was done on Markov textures, as suggested by Conners and Harlow,[24] in following combinations and the results are shown in Figure 2. The algorithm has been applied for detecting one texture pattern at a time.

**Figure 3:**
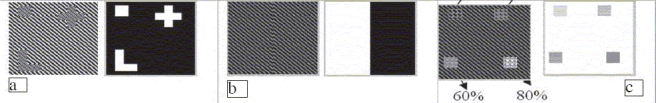
Markov textures (a) one of the patterns acting as base and the other pattern in different shapes, (b) two close and visually non-discriminable patterns placed side by side, (c) one pattern acting as base pattern and the other pattern distorted in the order of 20%, 40 %, 60%, and 80% distortion
- Two close and visually non-discriminable patterns placed side by side, as shown in Figure 3b
- One pattern acting as base and the other pattern distorted in terms of 20%, 40%, 60%, and 80% distortion. The results in Figure 3c show that the algorithm was able to segment the distorted pattern from the base pattern with 100% classification rate.

- One of the patterns acting as base and other pattern in different shapes, as shown in Figure 3a

### Application of algorithm on medical images

**Figure 4:**
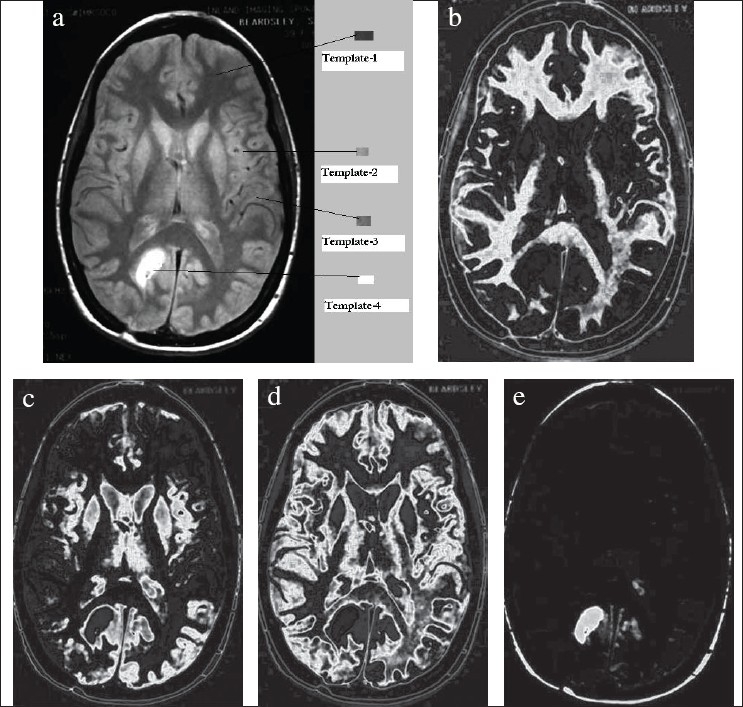
(a) Original brain CT image marked with templates, (b) Segment-1 corresponding to template-1, (c) Segment-2 corresponding to template-2, (d) Segment-3 corresponding to template-3, (e) Segment-4 corresponding to template-4

The software has been used for the segmentation of CT and MR images. (DICOM format medical images have been used; DICOM format supports medical images of both unit 8– and unit 16–type data [i.e., CT or MR image can be stored in both 8-bit and 16-bit formats]. Experimentation has been carried out on CT and MR images of different body parts and of different types [both unit 8 and unit 16 type], and promising results have been achieved.) Figure 4 shows the segmentation carried out on brain images using this software. The templates selected are shown in Figure 4a; corresponding to each template, the segments obtained are shown in Figures 4b-4e respectively.

**Figure 5:**
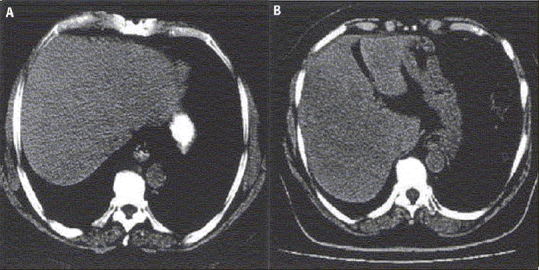
(A) shows normal liver, (B) shows hepatocellular liver

The same algorithm has been applied for the segmentation of abdomen CT image, hepatocellular carcinoma case. In case of liver images of hepatocellular carcinoma, we look for the change in textural pattern of carcinoma part as compared to normal liver [Figure 5A]. The textural pattern of hepatocellular part is coarse as compared to normal liver, a particular case of hepatocellular liver [Figure 5B]. Accordingly, the first- and second-order statistical features are calculated, as every textured pattern has different values of these features. The BAM-type ANN is able to classify them in their respective classes. A few values of these features taken for normal liver and hepatocellular liver are shown below:

Features′ values [mean, S.D., entropy, variance, contrast, correlation, energy, homogeneity, cluster tendency]

Normal liver = [0.1527, 0.1393, 0.4732, 0.5578, 0.6582, 0.0099, 0.0117, 0.5893, 0.4960]

Hepatocellular = [0.1700, 0.1468, 0.4511, 0.9062, 0.5931, 0.0085, 0.0109, 0.5321, 0.4181]

**Figure 6:**
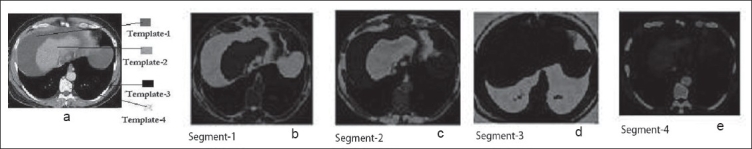
(a) Original abdomen CT image marked with templates, (b) Segment-1 corresponding to template-1, (c) Segment-2 corresponding to template-2, (d) Segment-3 corresponding to template-3, (e) Segment-4 corresponding to template-4

Figure 6 shows the abdomen CT to be segmented and the corresponding templates selected and the respective segmentation results. The results were found in agreement with the radiological evaluation. It can be concluded that this software would be of great help in the diagnosis and treatment planning. It would be an effective tool in auto-contouring for treatment planning and evaluation of radiotherapy outcome by measuring ROI.

**Figure 7:**
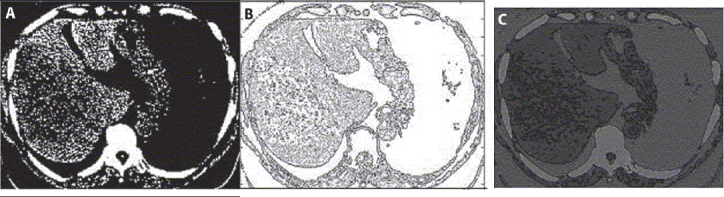
(A) Segmentation results using threshold technique; (B) Segmentation results using automated contouring techniques available in MATLAB; and (C) Segmentation results produced using present algorithm

Regarding the comparison of present algorithm with direct MATLAB base segmentation, the tools available in MATLAB for segmentation are based on the gray level–based segmentation techniques, for example, (i) simple gray level–based thresholding technique, (ii) graythresh, and (iii) imcontour commands. With graythresh command, at a time, only one type of ROI can be segmented. Further, the value of threshold is required to be determined from the histogram of the image. Again determination of exact value of threshold is a difficult task, particularly in multi-object image. In MATLAB using statistical toolbox, one can calculate some of the texture features; but for the segmentation purpose, one has to write own algorithm. Figures 7a and 7b show the segmentation results produced by using thresholding technique and automated contouring techniques available in MATLAB; and Figure 7c shows the segmentation results produced using the present algorithm [Figure 5B]. The quality of results produced using the present algorithm is much better than that by conventional techniques.

## Conclusion

In this paper, an algorithm for segmentation and classification of soft tissues on the basis of textural features of medical images based on BAM-type ANN specifically modified for image processing is established. The design of algorithm has been based on the concept that different types of soft tissues have different textural features, on the basis of which a clinician performs segmentation and classification; hence diagnosis could be made. Soft tissues such as cyst, hepatocellular carcinoma, hemangioma, in case of liver; and gray matter, white matter, cerebrospinal fluid (CSF) in case of brain — all have different textures in CT and MR images and therefore can be represented by their respective texture primitive, which along with its features can be used for analyzing the complete series of CT and MR images.

Although a variety of different neural network–based algorithms have been developed for texture-based segmentation and classification[25,26] with good classification accuracy, most of these texture classifier algorithms require extensive supervision and training; and their performance is affected in the presence of noise.

This modified BAM-type ANN-based algorithm has the following features:

- Supervision is required only for selecting texture primitives.
- It does not require training.
- It is able to provide results, particularly in the presence of noise and distortion.

Through this algorithm, it is shown that associative networks are capable of capturing both syntactical and statistical features and automatically segment the texture primitives that occur most frequently in an image; and further are capable of segmenting textures, which are not visually discriminable. From the experimental results, it can be deduced that the present algorithm is able to segment and classify close and visually non-discriminable pattern and performs well even under the presence of noise. The application of this algorithm has given 100% results on Markov textures and has shown promising results with medical images.

In the present paper, we have demonstrated the working of BAM-type ANN for template primitive-based segmentation, which is conceptually working well at present. However, the limitations of this algorithm are as follows: 1) Size of texture primitive may vary as per object required to be segmented and hence the computational time, which is a factor dependent on the size of texture primitive. 2) Correct labeling of texture primitive is required (the results reported on medical images may not be optimal as templates have been labeled on the estimation basis; further, results can be improved by taking mean template features selected at different locations). Despite these shortcomings, this algorithm is performing well for soft tissue characterization and segmentation; and further, the same can also be applied to other areas of image processing and pattern recognition.

The present algorithm can be modified by including other statistical features for increased reliability in segmentation and classification of still more complex medical images. As suggested by the reviewer, our future research will include the building up of ‘Look up’ Table (LUT) comprising of texture features corresponding to different soft tissue; LUT can serve as a problem-specific segmentation tool. Hence for each specific type of image (CT, MR) corresponding to different body parts, a separate LUT atlas can be built, involving more clinical trials.

### Appendix-I

**Figure 8:**
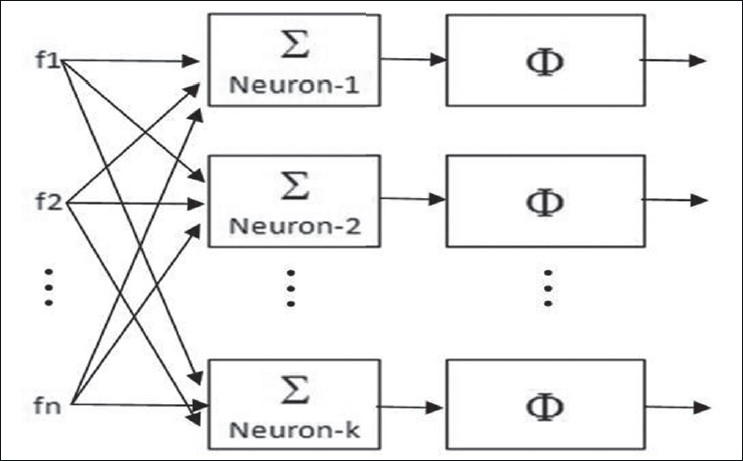
Architecture of BAM network

Architecture of BAM network: BAM network used in the present work is a single-layer network; architecture of same is shown in Figure 8. The number of neurons (k) used equals the number of objects (ROIs) to be segmented and classified; hence for each primitive template, there is one neuron. In the present example of segmentation of brain CT image, there are four primitive templates, and hence four neurons are used. The number of inputs to each neuron (n) equals the number of features in the input data. In present case, each neuron is having 17 inputs corresponding to 17 features. The feature vectors of primitive templates act as weight vectors of respective neurons. The transfer function used is threshold function (φ); the value of threshold corresponds to the desired level of confidence.

Algorithm for bidirectional associative memory–type ANN[22] is as fallows:

BAM(N,X,Y)/*Nis the number of pattern sets*//*(X,Y)are the pattern pair sets, whereX=(X1,X2,...,Xn)andY=(Y1,Y2,...,Yn)*//*Xi=(Xi1,Xi2,...,Xin)andYi=(Yj1,Yj2,...,Yjn)*/Step1:NormalizeX,Yfori←1toNXi(norm)=Xi‖Xi‖;Yi(norm)=Yi‖Yi‖end.

Step 2: Input A — the pattern vector to be segmented and classified, obtain its normalized equivalent

A(norm)=Ai‖Ai‖;

Step 3: Compute the inner product of A(norm) with xi(norm) where i = 1,2., … , N for j←1toNZi=A(norm).Xi(norm)end

Step 4: Apply threshold function ‘Φ’ on Z to obtain the correlation vector M, M = ^Φ^ (Z) =(m1,m2,....,mZ), where ^Φ^ is equal to one for maximum of

Z and zero elsewhere.

Step 5: Output Yk, where ‘k’ is such that mk = max (mi); where i =1, 2,., N.
